# Factors Influencing HPV Vaccination Uptake in Adolescents: Evidence to Guide Clinical Practice

**DOI:** 10.1111/wvn.70120

**Published:** 2026-02-18

**Authors:** Taylor Mullin, Diane Von Ah

**Affiliations:** ^1^ College of Nursing The Ohio State University Columbus Ohio USA; ^2^ Center for Healthy Aging, Self‐Management and Complex Care, College of Nursing The Ohio State University Columbus Ohio USA; ^3^ Co‐Leader of Cancer Control Program The Ohio State University James Comprehensive Cancer Center Columbus Ohio USA

**Keywords:** adolescents, HPV vaccination, public health, social determinants of health, systematic review, vaccine disparities

## Abstract

**Background:**

Human papillomavirus (HPV) is a major contributor to several preventable cancers. Although the HPV vaccine is recognized by the Centers for Disease Control and Prevention (CDC) as safe and effective, uptake among U.S. adolescents remains below optimal levels. Disparities in vaccination rates are shaped by both individual characteristics and social determinants of health (SDOH).

**Objective:**

To systematically review and synthesize the literature examining individual factors and social determinants of health associated with HPV vaccine initiation and completion among adolescents aged 9–18 years in the U.S.

**Methods:**

A systematic search was conducted in accordance with PRISMA guidelines, yielding 37 eligible studies from an initial pool of 2092 articles. The STROBE checklist was used to assess methodological quality, and the Levels of Evidence framework by Melnyk and Fineout‐Overholt guided appraisal of study strength.

**Results:**

Across included studies, initiation and completion rates averaged 47% and 40%, respectively. Key predictors of higher vaccine uptake included provider recommendation, health insurance coverage, urban residence, older age, and higher parental education. Disparities were most evident among adolescents living in rural areas and those from minority or low‐income backgrounds. Barriers reported in several studies included parental safety concerns and logistical challenges. Evidence regarding parental knowledge and attitudes was mixed: smaller studies suggested an influence, whereas the largest population‐based study reported no significant effect.

**Conclusion:**

Addressing HPV vaccination disparities requires a multifaceted approach, including improving healthcare access in underserved regions, strengthening provider–parent communication, and implementing policy interventions such as school‐based vaccination programs and state mandates. Normalizing HPV vaccination as part of routine adolescent care is essential for reducing HPV‐related cancer morbidity and mortality. These findings also have implications for catch‐up vaccination in young adults aged 15–26 and shared clinical decision‐making up to age 45, which remain important strategies for increasing protection across the lifespan.

## 1 Introduction

Human papillomavirus (HPV) infection poses a significant healthcare challenge, contributing substantially to the global disease burden and remaining a major public health concern (National Immunization Survey, 2022; World Health Organization 2022). As the most common sexually transmitted infection in the U.S., HPV is linked to the development of up to seven different cancers (Thompson et al. 2019). While cervical cancer is the most prevalent HPV‐related cancer in females, the virus also contributes to anal, vaginal, penile, rectal, oropharyngeal, and vulvar cancers (Aninye et al. 2021). In the U.S., an estimated 42.5 million individuals are currently infected with HPV, with approximately 48,000 new cases of HPV‐associated cancers diagnosed annually, impacting 26,280 women and 21,704 men (CDC 2023; Statista 2023).

HPV vaccines are recognized by the CDC as safe and effective for preventing HPV infections and associated cancers when administered on schedule (CDC 2023). The U.S. was the first country to license the HPV vaccine, initially recommending it for girls in 2006 and later extending recommendations to boys in 2010. Vaccines are typically administered during well‐child visits by primary care providers and are covered through private insurance or the federally funded Vaccines for Children program for underinsured or uninsured families (Akhatova et al. 2022). The CDC recommends routine HPV vaccination for adolescents aged 11–12. However, this target age group presents unique challenges due to public concerns about age appropriateness, perceived links to sexual activity, and safety—concerns less commonly associated with traditional childhood vaccines (Victory et al. 2019). In addition to routine vaccination, the CDC recommends catch‐up vaccination through age 26 for those not adequately vaccinated, and shared clinical decision‐making for adults aged 27–45 (CDC 2023). These extended recommendations highlight the importance of understanding uptake not only during adolescence but also among young adults, particularly those who may have delayed vaccination.

Despite vaccine availability and established efficacy, HPV vaccination coverage among U.S. adolescents remains below optimal levels. As of 2023, 78.5% of females and 75.1% of males aged 9–18 had initiated the vaccine series, but only 58.6% had completed the recommended two‐ or three‐dose schedule (CDC 2023; Statista 2023). Although these figures reflect progress, adolescents remain below national public health targets, underscoring persistent gaps in series completion. Furthermore, sociodemographic and geographic disparities persist, suggesting inequitable access to and uptake of HPV vaccination.

While existing research has examined certain predictors of HPV vaccine uptake, limited attention has been given to the intersection of individual‐level factors and broader social determinants of health (SDOH) that shape vaccination behaviors. Additionally, most studies focus on adolescents aged 13–17 years, consistent with the National Immunization Survey–Teen (CDC 2023), but this narrower age group overlooks important patterns among younger adolescents who initiate early or among those who delay vaccination. Moreover, findings on parental knowledge and attitudes remain mixed: some smaller studies suggest an influence, but the largest population‐based analyses reported no significant effect. These inconsistencies highlight the need for cautious interpretation when considering parental knowledge as a determinant of uptake.

The purpose of this literature review was to synthesize current evidence on individual characteristics and SDOH associated with HPV vaccine initiation and completion among adolescents aged 9–18. By expanding the scope beyond the commonly studied 13–17 age group, this review offers a more comprehensive analysis of vaccination behaviors across developmental stages and enhances alignment with CDC guidelines recommending initiation at ages 11–12. Evaluating both early initiation and delayed completion provides a nuanced understanding of where gaps exist in the vaccination process. A comprehensive examination of HPV vaccine uptake—including initiation, completion, and timeliness—can help identify intervention points across the continuum of care. Through this synthesis, the review also underscores how adolescent‐focused findings connect with catch‐up vaccination efforts in older populations, ultimately informing equity‐driven, targeted strategies to improve coverage and reduce HPV‐related cancer burden.

## 2 Methods

To examine the factors influencing HPV vaccine uptake among adolescents, a systematic literature review was conducted using rigorous and structured methods. The review aimed to identify peer‐reviewed studies that evaluated individual characteristics and SDOH associated with HPV vaccine initiation and completion among adolescents aged 9–18 years. The search strategy, study selection process, and data extraction procedures were guided by established best practices for systematic reviews.

### 2.1 Search Strategy

A comprehensive literature review was conducted using the PubMed, CINAHL, Scopus, and EMBASE databases. The search filters were restricted to English‐language studies that included adolescents aged 9–18 years of any sex. Search terms and filters targeted SDOH as independent variables, guided by the Healthy People 2030 framework. The primary outcomes of interest were HPV vaccination initiation, completion, uptake, and adherence. For clarity, initiation was defined as receipt of at least one HPV vaccine dose, completion as receipt of the full two‐ or three‐dose series (depending on age at initiation), uptake as overall coverage regardless of series status, and adherence as receiving all doses within the recommended time frame.

**FIGURE 1:**
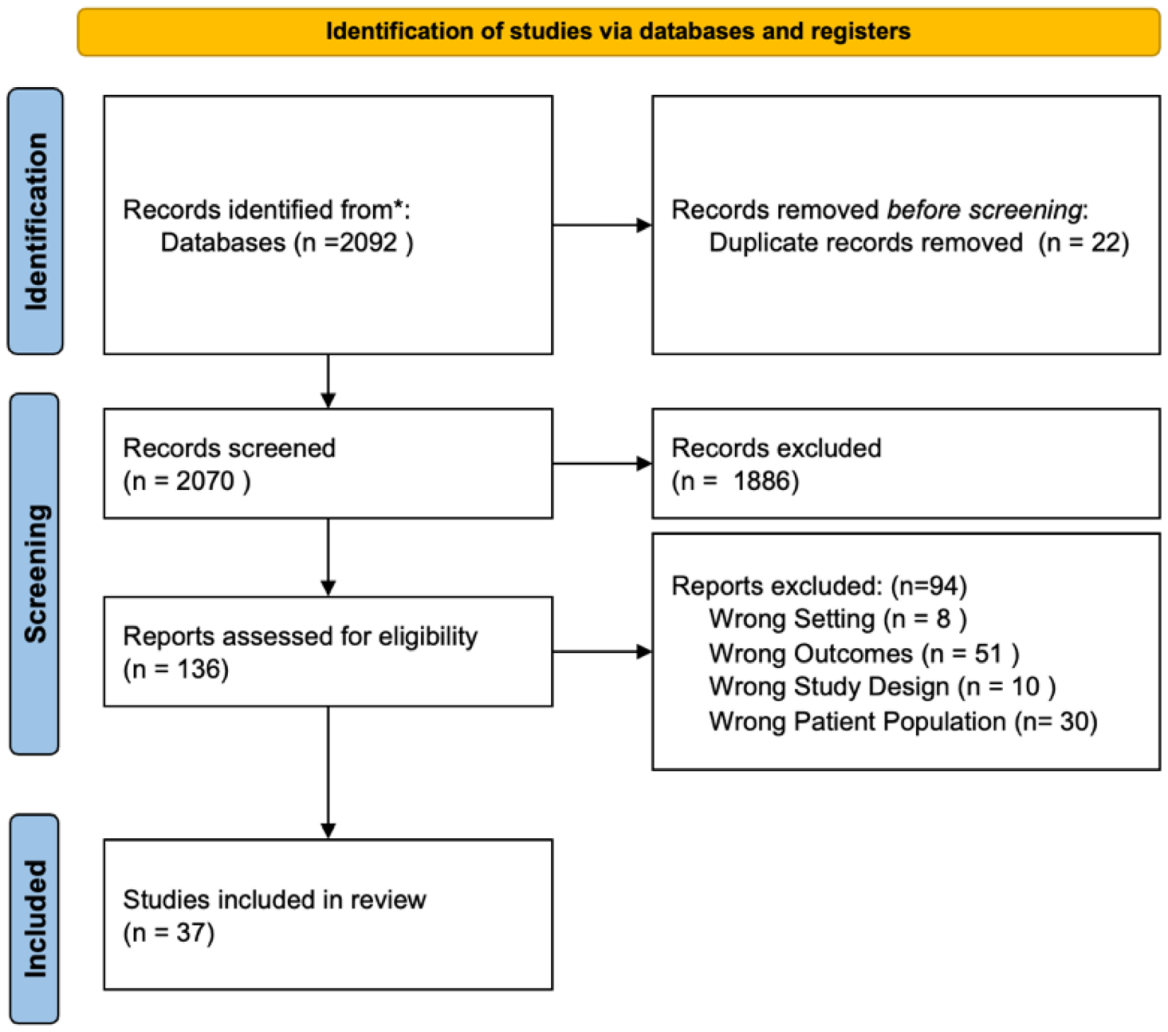
PRISMA decision tree for study selection.

To ensure consistency and relevance to U.S. health policy, insurance systems, and vaccine delivery infrastructure, only studies conducted within the U.S. were included. While other countries may have comparable HPV vaccination schedules, access to vaccines and financial coverage mechanisms differ significantly across nations, which could affect generalizability. The initial search across all databases yielded 2092 articles. The study protocol was registered on PROSPERO prior to conducting the search. This review is reported in accordance with the Preferred Reporting Items for Systematic Reviews and Meta‐Analyses (PRISMA) 2020 guidelines to ensure transparency and completeness in reporting (Figure 1).

### 2.2 Selection Criteria

Studies were included if they met specific inclusion criteria. The target population was adolescents aged 9–18 years, and only studies conducted within the U.S. were considered to ensure cultural and healthcare system relevance. Articles were limited to those published in English to maintain consistency in data interpretation and accessibility.

Eligible studies were required to examine SDOH variables within the framework of the Healthy People initiatives and to report HPV vaccination outcomes, including initiation, completion, uptake, or adherence. Manuscripts were excluded if they addressed vaccinations other than HPV, included populations outside the specified age range, or used non‐empirical designs (e.g., editorials, commentaries, reviews without original data). Studies that did not report HPV vaccination outcomes were excluded, as were those conducted outside the U.S. to maintain focus on the specific U.S. healthcare context.

A total of 22 duplicate articles were removed using the Covidence electronic screening system. Following the title and abstract review, 1886 articles were excluded based on predefined eligibility criteria (Figure 1). Full‐text review was conducted for 136 peer‐reviewed articles to determine their relevance. The inclusion and exclusion criteria were applied systematically to ensure alignment with the study purpose.

### 2.3 Data Extraction

Two independent reviewers extracted data from the included studies using a standardized form. Extracted variables included study year, sample size, age range, HPV vaccination outcomes, SDOH variables, and study design. In accordance with the Healthy People 2030 framework, variables were categorized as either individual‐level factors (e.g., age, sex, parental attitudes) or SDOH (e.g., income, education, insurance coverage, geographic location). Initial inter‐rater agreement was 95%. Discrepancies were resolved through collaborative review and consensus discussions, and all final inclusion decisions were made jointly.

### 2.4 Quality Assessment and Level of Evidence

To ensure a rigorous and systematic evaluation of the selected studies, this research employed both the Strengthening the Reporting of Observational Studies in Epidemiology (STROBE) checklist (von Elm et al. 2007). The STROBE checklist was used to guide the reporting of observational studies, including cohort, case‐control, and cross‐sectional designs to ensure transparency and completeness in the presentation of study methods and findings. The checklist evaluated critical elements such as study design, participant selection, data collection methods, bias, statistical analysis, and interpretation of findings. All studies were included in the STROBE checklist. The checklist provided a systematic means to evaluate the rigor and reliability of these studies and identify potential limitations.

Melnyk and Fineout‐Overholt's levels of evidence framework was utilized to classify the strength of the included studies (Melnyk and Fineout‐Overholt 2023). This hierarchy categorizes evidence from Level 1 to Level VII. The reviewed studies predominantly fell under Level IV evidence, reflecting their observational nature while offering insights into associations between individual and SDOH and HPV vaccination coverage. Additionally, some quasi‐experimental studies (Level III) were included, particularly those assessing the impact of interventions.

## 3 Results

A total of 2092 articles were identified through the initial search, and 37 studies met the inclusion criteria for this review. The screening and selection process is presented in the PRISMA flow diagram (Figure 1). These studies collectively explored individual characteristics and SDOH as predictors of HPV vaccine initiation and completion among adolescents. The findings represent a narrative synthesis rather than pooled statistical estimates.

### 3.1 Study Characteristics

Of the 37 included studies, 28 (75.7%) were secondary analyses of existing datasets, 6 (16.2%) were retrospective cohort studies, and 3 (8.1%) employed cross‐sectional survey designs. Approximately half (*n* = 18) utilized the National Immunization Survey–Teen (NIS‐Teen) data, though each study examined different timeframes, subpopulations, or predictors. Overall, 25 studies (67.6%) examined both initiation and completion, while others focused specifically on uptake (8/37; 21.6%), initiation only (1/37; 2.7%), completion only (1/37; 2.7%), adherence (1/37; 2.7%), or coverage (1/37; 2.7%). A detailed summary of study characteristics, including methods, populations, and outcomes, is provided in Table 1.

**TABLE 1:** Key elements included in the 37 articles documents not only their study but level of evidence and strobe.

Author, year	Year of study/dataset	Methods	Age range and sex	*N*	HPV vaccination outcome variables	Level of evidence	STROBE Checklist Number and Quality Ranking
Adjei Boakye et al. (2023)	2015–2020 in Southern Illinois	Retrospective	11–17 M & F	9351	Initiation and completion	IV	1, 2, 6, 10, 12, 16; High
Adjei Boakye et al. (2017)	2014 NIS‐teen	Secondary analysis of cross‐sectional data	13–17 M & F	20,827	Initiation and completion	IV	2, 7, 9, 11, 13, 17; High
Agawu et al. (2015)	2009–2013 large primary care network	Retrospective Cohort	11–18 M	58,757	Initiation and completion	IV	3, 5, 8, 10, 14, 18; Medium
Bednarczyk et al. (2019)	2016 NIS‐teen	Secondary analysis of cross‐sectional data	13–17 M & F	20,475	Initiation and completion	IV	3, 6, 8, 10, 14, 16; High
Bhatta and Phillips (2015)	2012 Middle and High School Youth risk Behavior Surveillance System Survey	Secondary analysis	11–18 M & F	1299	Uptake^a^	IV	1, 5, 7, 10, 13, 17; High
Clark et al. (2016a)	2012	Cross‐sectional survey	9–17 M & F	1653	Initiation and completion	IV	3, 6, 9, 12, 15, 18; High
Clark et al. (2016b)	2012	Cross‐sectional survey	9–17 M & F	791	Completion	IV	2, 5, 8, 11, 14, 17; High
Cullen et al. (2014)	2009–2012 Immunization Information System	Secondary analysis	11–18 M & F	2,900,000	Initiation and completion	IV	1, 4, 7, 10, 12, 16; High
Ejezie et al. (2024)	2019–2021 NIS‐teen	Secondary analysis of cross‐sectional data	13–17 M & F	38,128	Uptake^a^	IV	2, 6, 9, 12, 14, 18; High
Fuchs et al. (2016)	2011–2013 University of Texas Medical Branch HPV vaccination survey	Secondary analysis	9–17 M & F	350	Initiation and completion	IV	2, 5, 7, 11, 13, 17; High
Goodman et al. (2023)	2017–2020 NIS teen	Retrospective cohort	13–17 M & F	19,575	Intitation and completion	IV	1, 4, 7, 10, 13, 17; High
Henry et al. (2017)	2012 & 2013 NIS teen	Secondary analysis of cross‐sectional data	13–17 M	19,518	Initiation and completion	IV	2, 5, 9, 11, 13, 17; High
Jeyarajah et al. (2016)	2008–2013 NIS teen	Secondary analysis of cross‐sectional data	13–17 F	33,707	Initiation and completion	IV	1, 4, 7, 10, 12, 16; High
Johnson et al. (2017)	2013 NIS teen	Secondary analysis of cross‐sectional data	13–17 M & F	18,264	Vaccine adherence^b^	IV	2, 5, 7, 10, 13, 17; High
Kepka et al. (2015)	2013 Salt Lake City, Utah 38 item survey	Survey	11–17 M & F	67	Uptake^a^	IV	1, 4, 8, 11, 13, 17; High
Krakow et al. (2017)	2014 NIS teen	Secondary analysis of cross‐sectional data	13–17 M & F	12,742	Initiation	IV	3, 6, 9, 12, 14, 18; High
Lai et al. (2016)	2012 NIS data	Secondary analysis of cross‐sectional data	13–17 M & F	1291	Initiation and completion	IV	1, 5, 8, 10, 14, 17; High
Lu et al. (2015)	2013 NIS teen	Secondary analysis of cross‐sectional data	13–17 M & F	9554	Coverage and uptake^c^	IV	2, 6, 8, 10, 13, 17; High
Lu et al. (2018)	2015 NIS teen	Secondary analysis of cross‐sectional data	13–17 M & F	21,875	Initiation and completion	IV	2, 6, 8, 11, 13, 17; High
Mansfield et al. (2021)	2018–2019	Retrospective chart review with a cohort design	11–14 M & F	324	Series completion and timely series completion^d^	IV	3, 6, 9, 11, 14, 18; High
Munn et al. (2019)	2013 WAIIS data	Secondary analysis	13–18 M & F	21,197	Uptake and completion	IV	2, 6, 9, 12, 13, 17; High
Pourebrahim et al. (2021)	2008–2018 WAIIS data	Secondary analysis	11–12 M & F	402,547	Uptake^a^	IV	2, 5, 7, 11, 13, 17; High
Pruitt et al. (2022)	2013–2017 NIS teen	Secondary analysis of cross‐sectional data	13–17 M & F	63,299	Initiation and completion	IV	2, 6, 9, 11, 13, 17; High
Rahman et al. (2017)	2013 NIS teen	Secondary analysis of cross‐sectional data	13–17 F	9403	Initiation and completion	IV	2, 6, 9, 12, 14, 18; High
Rahman et al. (2015a)	2011 NIS teen	Secondary analysis of cross‐sectional data	13–17 M & F	39,839	Initiation and completion	IV	2, 5, 8, 10, 13, 17; High
Rahman et al. (2015b)	2012 NIS teen	Secondary analysis of cross‐sectional data	13–17 F	4548	Initiation and completion	IV	3, 6, 9, 12, 14, 18; High
Reiter et al. (2014)	2010–2012 NIS teen	Secondary analysis of cross‐sectional data	13–17 M	4238	Initiation and completion	IV	2, 5, 8, 11, 13, 17; High
Staples et al. (2021)	2018–2018 VIIS	Secondary analysis	11–17 M & F	13,388	Initiation and completion	IV	3, 6, 9, 12, 15, 18; High
Staras et al. (2021)	2006–2019 Florida state online tracking system	Secondary analysis	9–17 M & F	262,727	Initiation and completion	IV	3, 6, 9, 11, 14, 18; High
Swiecki‐Sikora et al. (2019)	2012–2013 NIS teen	Secondary analysis of cross‐sectional data	13–17 M & F	38,705	Initiation and completion	IV	2, 6, 9, 12, 13, 17; High
Teplow‐Phipps et al. (2016)	New York City Department of Health and Mental Hygiene Bureau of Immunization's Immunization information system	Retrospective analysis	9–18 M & F	1,494,767	Initiation and completion	IV	3, 6, 9, 12, 14, 18; High
Thompson et al. (2020)	2011–2017 NIS teen	Secondary analysis of cross‐sectional data	13–17 M & F	145,153	Uptake^a^	IV	2, 6, 8, 11, 13, 17; High
Torres et al. (2022)	2015–2018 NIS teen	Secondary analysis of cross‐sectional data	13–17 M & F	81,899	Initiation and completion	IV	3, 6, 9, 12, 15, 18; High
Varman et al. (2018)	2015 Outpatient Pediatric Clinic data in Omaha, Nebraska	Retrospective assessment	9–17 M & F	3393	Initiation and completion	IV	2, 5, 8, 11, 13, 17; High
White et al. (2023)	2011–2021 NIS teen	Secondary analysis of cross‐sectional data	13–17 M & F	220,806	Initiation and completion	IV	3, 6, 9, 12, 14, 18; High
Yankey et al. (2020)	2015–2017 NIS teen	Secondary analysis of cross‐sectional data	13–17 M & F	63,299	Initiation and completion	IV	3, 6, 9, 12, 14, 18; High
Yoo et al. (2020)	2008–2016 NIS teen	Secondary analysis of cross‐sectional data	13–17 M & F	86.705	Uptake^a^	IV	3, 6, 9, 12, 14, 18; High

^a^ Uptake is defined as at least 1 HPV vaccine received.

^b^ Vaccine Adherence is defined by initiation, completion, and intent to initiate.

^c^ Coverage refers to > 1 dose of HPV vaccination. Uptake refers to both ≥ 1 dose and ≥ 3 doses.

^d^ Timely series completion (defined as series completion within 14 months).

### 3.2 Initiation

Initiation of the HPV vaccine series was examined in 31 of the 37 studies (84%). Across these studies, age consistently emerged as significant, with younger adolescents—particularly those aged 11–12—being more likely to initiate vaccination than older adolescents. Geographic location was another critical determinant; of the 24 studies assessing urban–rural differences, 19 found that adolescents in rural settings were less likely to initiate vaccination than those in urban areas.

Sex at birth was examined in 30 studies (81%), with 26 reporting that females were more likely than males to initiate vaccination. Race and ethnicity were evaluated in 32 studies, with 28 reporting disparities. Non‐Hispanic White adolescents generally had higher initiation rates than minority groups, though some studies found higher initiation among Hispanic girls (e.g., Yoo et al. 2020).

Socioeconomic status also influenced initiation. Health insurance coverage was assessed in 31 studies, and 27 identified significant positive associations. Household income and parental education were analyzed in 31 and 32 studies, respectively, with 26 in each reporting significant associations.

**FIGURE 2:**
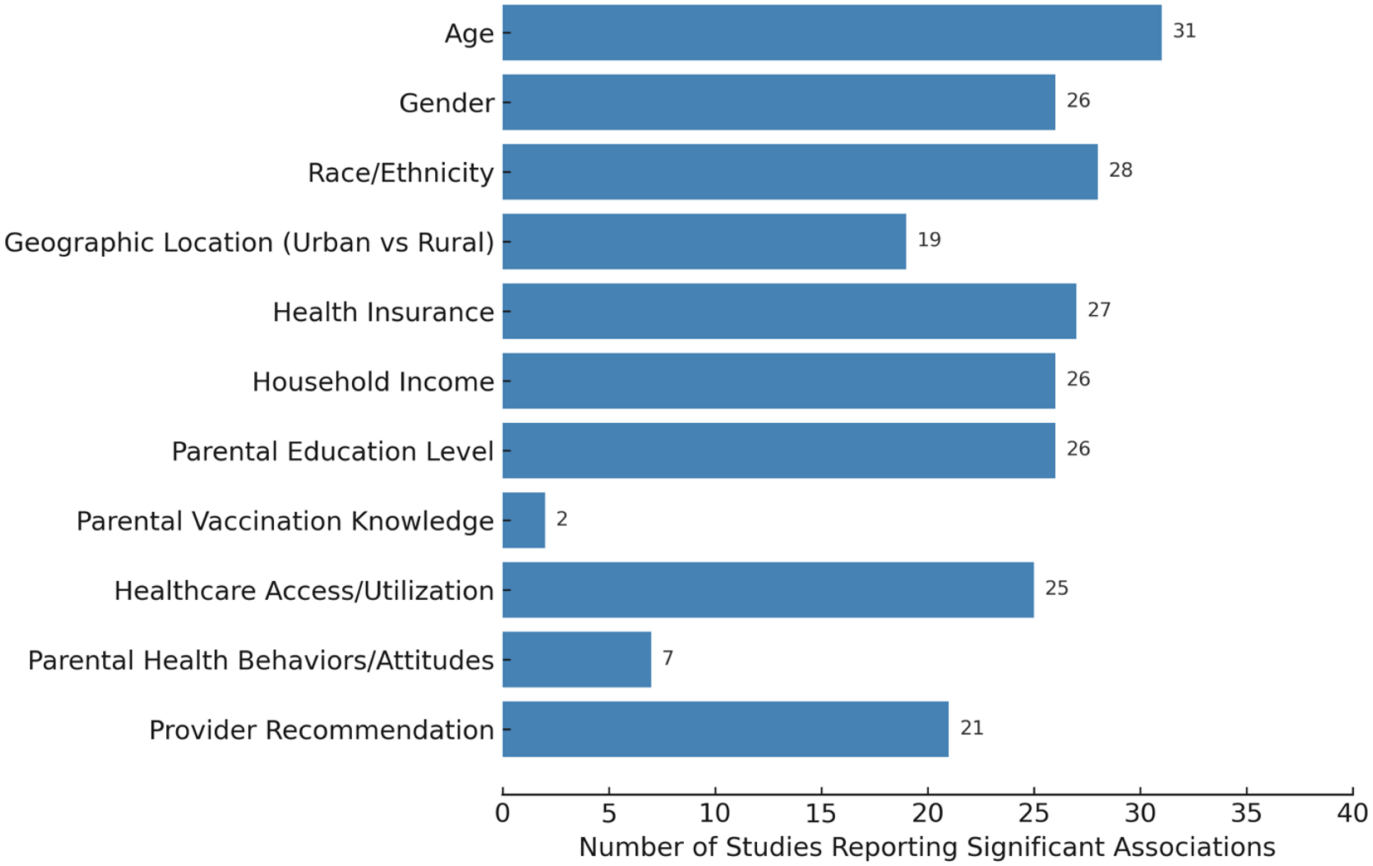
Frequency of social determinants of health associated with HPV vaccination initiation (*n* = 37).

Healthcare access and provider recommendation were among the strongest predictors. Of 28 studies examining healthcare access, 25 reported significant associations. All 21 studies evaluating provider recommendation identified a positive effect, underscoring the influence of clinician endorsement. Findings related to parental knowledge (3 studies, 2 significant) and parental attitudes (10 studies, 7 significant) were mixed, with the largest population‐based analysis reporting no significant effect. Thus, while these factors contribute to understanding hesitancy, their influence appears less consistent than structural and provider‐level determinants. Table S1 and Figure 2 summarize 37 studies that investigated factors associated with HPV vaccine initiation, organized by SDOH domains.

### 3.3 Completion

Completion of the HPV vaccine series was addressed in 29 studies (78.4%). As with initiation, age was consistently associated with completion, with younger adolescents more likely to complete the series once started. Geographic disparities were reported in 15 of 20 studies analyzing location, with rural adolescents showing lower completion rates.

Sex at birth was examined in 29 studies, with 26 finding females more likely to complete. Race and ethnicity were significant in 26 of 31 studies, indicating persistent disparities among racial/ethnic minority groups.

Insurance coverage and income were strong predictors of completion, with 25 of 29 studies linking insurance to higher completion and 26 of 30 reporting positive associations with higher income. Parental education (26/32) and healthcare access (26/29) were also significant. All 23 studies assessing provider recommendation found a positive association with series completion, highlighting the importance of clinical communication.

**FIGURE 3:**
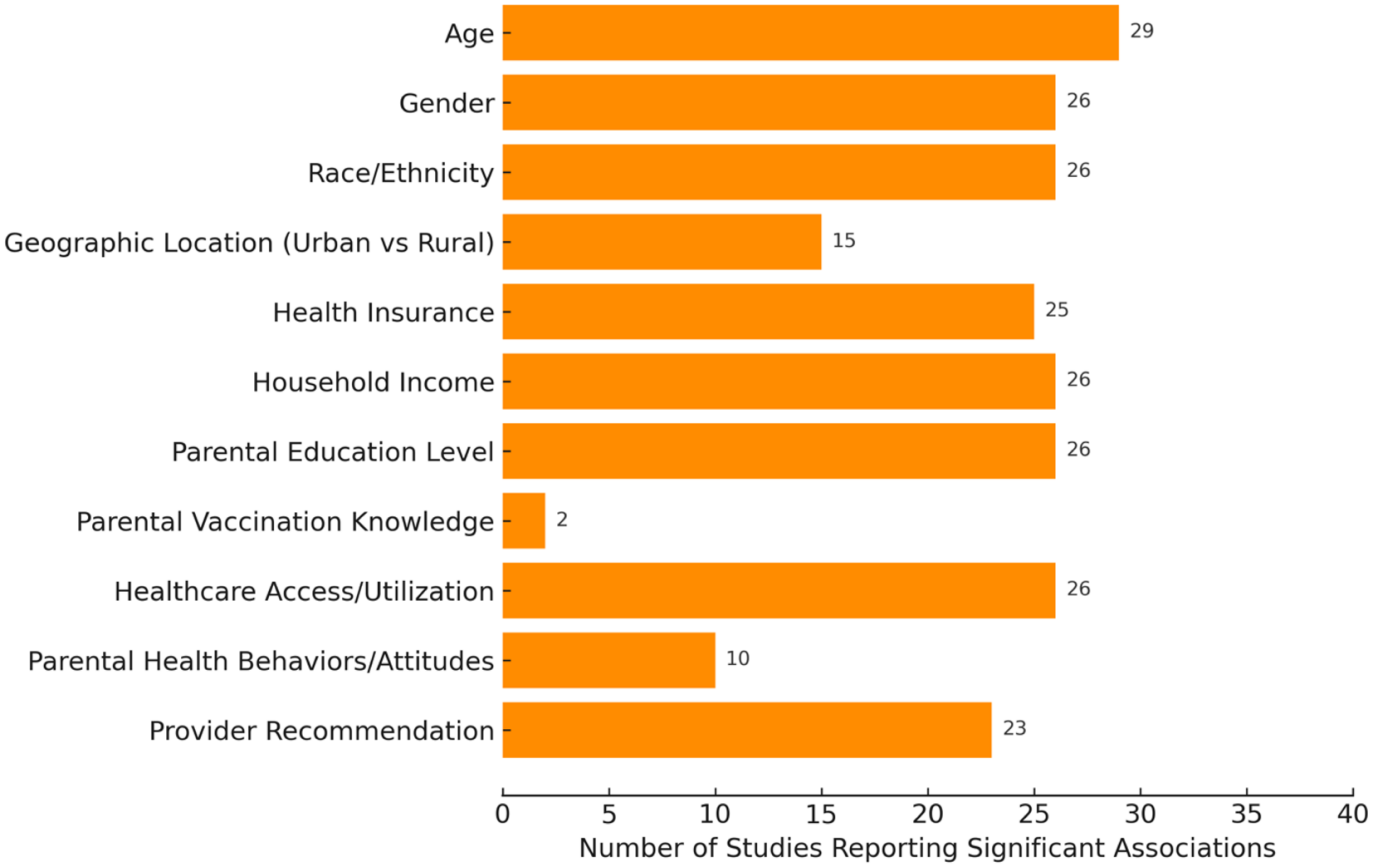
Frequency of social determinants of health associated with HPV vaccination completion (*n* = 37).

Parental attitudes and knowledge were less commonly assessed (10/37 and 2/37 studies, respectively), and findings were inconsistent. This underscores that, although attitudes may influence completion in some contexts, structural and provider‐related factors appear more consistently predictive. Table S2 and Figure 3 provide a synthesis of these findings across the same SDOH domains as Table S1.

### 3.4 Rural–Urban and Sociodemographic Disparities

Significant rural–urban disparities were evident, with rural adolescents, particularly those in Illinois, demonstrating lower vaccination rates than their urban counterparts (Adjei Boakye et al. 2023). Contributing factors included limited healthcare facility access, fewer healthcare providers, and lower levels of health education in rural areas. Other studies highlighted the importance of sociodemographic factors such as race, income, and parental education on vaccination rates. For example, Agawu et al. (2015) found that adolescent males from higher‐income families and those with highly educated parents were more likely to initiate the HPV vaccine. Clark et al. (2016a) reported that adolescents with higher income, better‐educated parents were also more likely to complete the HPV vaccine series.

The literature highlights the interconnected impact of race, income, and geographic location on HPV vaccination rates, with these factors often compounding disparities in initiation and completion. Adolescents from minority and low‐income backgrounds were found to have lower vaccination rates, as observed by Pourebrahim et al. (2021), who noted these disparities in urban and rural populations. Similarly, Yankey et al. (2020) identified significant racial disparities in the Mississippi Delta, where African American adolescents were less likely to be vaccinated than their white peers. National studies further support these findings; Yoo et al. (2020) reported that while Hispanic and African American girls were more likely to initiate the vaccine series, they were less likely to complete it, a trend also documented by Lu et al. (2015) among lower‐income and rural male adolescents. Additionally, Staples et al. (2021) examined geospatial disparities in Virginia, linking lower vaccination rates to income and geographic location, emphasizing the need for targeted interventions addressing these overlapping barriers.

Geographic disparities in HPV vaccination rates were evident, with certain states and regions consistently reporting lower rates. For example, Pourebrahim et al. (2021) highlighted significant variations within Washington State, where specific areas had persistently lower rates. Meanwhile, White et al. (2023) found that overall vaccination rates improved over time, but regional disparities remained. Torres et al. (2022) observed that states with policies allowing adolescents to consent to HPV vaccination without parental approval had higher vaccination rates. Yoo et al. (2020) showed that state‐level policies promoting HPV vaccination, along with increased healthcare access, were associated with higher vaccination rates among female adolescents. These findings underscore the influence of local healthcare infrastructure, state policies, and societal attitudes on vaccination uptake. Pruitt et al. (2022) discussed missed vaccination opportunities, stressing the importance of timely vaccinations to reduce disparities. Thus, addressing the interplay of race, income, geographic location, and state‐level policies is essential to reducing disparities in HPV vaccination rates and improving timely uptake across diverse populations.

### 3.5 Other Considerations

#### 3.5.1 Provider Recommendations

Provider recommendation consistently emerged as the strongest predictor of both initiation and completion. Studies highlighted the importance of strong, clear, and tailored messaging. For example, Rahman et al. (2015b) found that strong provider recommendations increased initiation and completion, while Clark et al. (2016a) emphasized the role of consistent messaging. Teplow‐Phipps et al. (2016) demonstrated that gender‐specific recommendations further enhanced uptake. However, discrepancies between provider‐reported and parent‐reported vaccination rates (Adjei Boakye et al. 2017) suggest recall bias which may complicate measurement.

#### 3.5.2 Parental Perceptions and Beliefs

Parental concerns about safety and necessity were frequently cited as barriers, though findings were mixed. Some studies (e.g., Clark et al. 2016b; Fuchs et al. 2016) linked safety concerns and low perceived need to lower uptake, while others found no significant association in larger datasets. These results indicate that, while parental beliefs may influence individual cases, structural and provider‐related factors are more consistently impactful at the population level.

#### 3.5.3 Challenges in Vaccine Series Completion

Completion was influenced by logistical barriers, such as multiple appointments and follow‐up requirements (Clark et al. 2016b). Adolescents with access to school‐based health centers were more likely to complete the series (Munn et al. 2019), though not all centers directly administered vaccines. Early initiation also supported completion: Bednarczyk et al. (2019) reported that starting at younger ages increased the likelihood of finishing the series. Persistent disparities remained, however, with lower‐income and rural adolescents less likely to complete despite strong provider support (Mansfield et al. 2021; Rahman et al. 2017).

#### 3.5.4 Time Trends and Community‐Based Interventions

Several U.S. studies have examined HPV vaccination trends over time, with findings consistently showing gradual improvements in vaccination rates across adolescent populations. However, these gains have not been equitably distributed. Persistent disparities remain based on race, ethnicity, socioeconomic status, and geographic location, reinforcing the need for sustained and targeted public health interventions.

Cullen et al. (2014) reported modest increases in HPV vaccine initiation over time but noted that these improvements were not equally observed across all demographic groups. Socioeconomic barriers and limited access to preventive care services continued to affect vaccine uptake in underrepresented populations. Similarly, Jeyarajah et al. (2016) observed that while younger adolescent cohorts were more likely to initiate HPV vaccination at the recommended age, racial and socioeconomic disparities remained consistent across survey years. These findings suggest that although national awareness and provider recommendation patterns may have improved over time, systemic barriers continue to hinder equitable vaccine distribution.

Some studies highlight specific patterns within racial and ethnic groups. For instance, Reiter et al. (2014) identified early adoption of the HPV vaccine among Hispanic adolescent males, suggesting that culturally tailored messaging and family‐based decision‐making may contribute to higher vaccine acceptance within certain communities. This finding underscores the importance of understanding sociocultural contexts when developing public health strategies. In response to ongoing disparities, community‐based interventions have demonstrated promise in improving vaccination rates. Varman et al. (2018) found that localized efforts, such as school‐based clinics, community outreach, and partnerships with trusted organizations, significantly increased HPV vaccine initiation in underserved areas. Similarly, Staras et al. (2021) linked increased county‐level vaccination rates to reduced HPV‐related cancer incidence, providing evidence of the long‐term population health impact of localized public health efforts.

Thompson et al. (2019) examined HPV vaccination behaviors and found that individual characteristics alone could not account for persistent disparities in uptake. Their findings reinforced the influence of SDOH, highlighting the need to address structural and community‐level factors—such as insurance coverage, provider availability, and neighborhood context—through coordinated public health and policy interventions. Together, these studies demonstrate that although HPV vaccination rates in the U.S. have improved over time, structural inequities continue to shape who gets vaccinated and when. The evidence supports the need for culturally responsive, community‐driven, and system‐level interventions to reduce disparities and improve vaccine coverage nationwide.

## 4 Discussion

This literature review highlights the individual and social determinants that influence HPV vaccination initiation and completion among adolescents aged 9–18. Consistent with prior research, uptake is shaped by a constellation of factors, including sociodemographic characteristics, provider interactions, parental perceptions, geographic location, and policy environments (Rahman et al. 2015a; Pourebrahim et al. 2021; Lu et al. 2015). These findings emphasize the need for targeted, multi‐level interventions to reduce disparities, particularly among underserved and minority populations (Swiecki‐Sikora et al. 2019; Adjei Boakye et al. 2023).

### 4.1 Individual and Sociodemographic Influences on HPV Vaccination

Socioeconomic factors—including income, parental education, and insurance coverage—emerged as consistent predictors of uptake. Adolescents from higher‐income households and those with more educated parents were more likely to initiate and complete vaccination. Disparities by race and ethnicity, particularly lower completion among African American and Hispanic adolescents, reflect systemic barriers related to healthcare access and trust (Yankey et al. 2020). These inequities are compounded in rural, low‐income communities, where adolescents often face multiple intersecting barriers. Addressing these disparities requires culturally tailored interventions, such as community health worker programs or partnerships with trusted organizations, to improve awareness and access (Varman et al. 2018).

### 4.2 Role of Healthcare Providers in Improving Vaccination Rates

Provider recommendation consistently emerged as the strongest predictor of both initiation and completion. Studies demonstrated that clear, culturally sensitive recommendations significantly increase uptake (Bednarczyk et al. 2019; Krakow et al. 2017). Discrepancies between parent‐ and provider‐reported vaccination rates suggest communication gaps that may hinder completion (Adjei Boakye et al. 2017). Expanding access to school‐based health centers (SBHCs), which facilitate timely vaccinations, could help mitigate disparities. Tailored, gender‐sensitive recommendations may also improve uptake among adolescent males, who consistently lag behind females (Agawu et al. 2015; Gilkey et al. 2012).

### 4.3 Parental Beliefs and the Necessity of Addressing Safety Concerns

Parental perceptions of safety and necessity remain relevant but less consistent predictors than provider recommendation or socioeconomic factors. While smaller studies linked safety concerns to non‐completion (Clark et al. 2016b; Fuchs et al. 2016), larger population‐based analyses reported no significant associations. These findings suggest that, although parental beliefs may influence individual decisions, structural and provider‐related factors are more reliably predictive at the population level. Public health campaigns should continue addressing safety misconceptions and normalizing HPV vaccination as part of routine adolescent care (Johnson et al. 2017).

### 4.4 Geographic and Policy‐Driven Variations in Vaccination Rates

Geographic and policy environments strongly shaped vaccination rates. Adolescents in urban areas and in states with policies supporting vaccination (e.g., adolescent consent laws, school‐entry mandates) demonstrated higher uptake (Torres et al. 2022; Thompson et al. 2020). Persistent rural–urban gaps reflect limited provider availability and infrastructure in rural communities (Aninye et al. 2021). Proposed solutions include expanding mobile vaccination units, telehealth, and provider incentives in underserved regions. State‐level policies represent an important structural lever to increase uptake, particularly where local healthcare infrastructure is limited.

### 4.5 Barriers to Series Completion and the Importance of Follow‐Up Interventions

While initiation rates are a positive step, the significant drop in HPV vaccine series completion underscores the importance of sustained follow‐up. Adolescents who initiate the series at a younger age are more likely to complete it, supporting early vaccination efforts in line with Advisory Committee on Immunization Practices (ACIP) guidelines. However, logistical barriers such as multiple required visits and insufficient follow‐up support continue to challenge completion rates, particularly among low‐income and rural adolescents (Rahman et al. 2017; Goodman et al. 2023). Initiatives that integrate reminder systems, such as text or email notifications, can support series completion by helping families track vaccination schedules. Additionally, policymakers could explore developing reimbursement models to incentivize providers for completing vaccination series with underserved populations, addressing logistical challenges tied to follow‐up appointments. Expanding mobile public health initiatives and school‐based vaccination programs could also be pivotal in improving access (Mansfield et al. 2021; Lu et al. 2018). While vaccine hesitancy remains a contemporary challenge, the historical success of school‐based immunization campaigns, during which large numbers of children were routinely vaccinated, demonstrates the potential effectiveness of implementing similar strategies to improve vaccination rates today. Re‐establishing these community‐centered approaches could help overcome barriers and ensure that adolescents, particularly in underserved areas, receive timely and complete HPV vaccinations. Expansion of school‐based health centers and other low‐barrier healthcare settings for adolescents could also address this issue by providing convenient, consistent access to both vaccination initiation and series completion (White et al. 2023). Key findings from this review have been synthesized into actionable recommendations in Table 2 to guide healthcare providers and policymakers.

**TABLE 2:** Linking evidence to action.

Evidence‐based finding	Recommended action
Strong provider recommendations significantly increase HPV vaccine initiation and completion	Implement provider training programs focused on delivering clear, culturally sensitive HPV vaccine messages
Adolescents from low‐income, rural, and minority backgrounds have lower vaccination completion rates	Expand access to school‐based health centers and mobile clinics in underserved and rural communities
Parental safety concerns and misinformation contribute to vaccine hesitancy and non‐completion	Develop and disseminate targeted educational materials addressing HPV vaccine safety and benefits
State policies allowing adolescent self‐consent are linked to higher vaccination rates	Advocate for policy reforms to permit adolescent consent for HPV vaccination in states where not allowed
Early initiation (ages 11–12) leads to higher series completion rates	Promote HPV vaccination during routine well‐child visits starting at ages 9–12
Missed follow‐up appointments hinder series completion, especially in disadvantaged groups	Establish reminder systems (e.g., texts, calls) and reimbursement incentives for providers who ensure completion

Abbreviation: HPV, human papillomavirus.

### 4.6 Strengths, Limitations and Future Research Directions

This literature review has several notable strengths. First, it includes a broad age range (9–18 years), enabling a comprehensive examination of HPV vaccination behaviors across developmental stages (White et al. 2023). Unlike many studies that focus solely on initiation, this review also addresses completion and adherence, offering a holistic view of vaccination uptake (Goodman et al. 2023; Rahman et al. 2017). Additionally, the systematic approach used, guided by PRISMA, and incorporating tools like the STROBE checklist and Melnyk and Fineout‐Overholt's Levels of Evidence framework, ensured a rigorous evaluation of the quality and relevance of the included studies.

Despite these strengths, several limitations should be acknowledged. The types of studies included, predominantly retrospective and cross‐sectional designs, limit the ability to establish causal relationships (von Elm et al. 2007). Geographic focus was another constraint, as many studies were region‐specific, reducing the generalizability of findings. Variability in the definitions of vaccination outcomes, such as initiation and completion, further complicates cross‐study comparisons (Swiecki‐Sikora et al. 2019; Staples et al. 2021). Furthermore, the reliance on self‐reported data in numerous studies introduces potential recall bias and inaccuracies, particularly when compared to provider‐reported or electronic health records (Adjei Boakye et al. 2017; Aninye et al. 2021).

Future research should address these limitations to advance the field. Standardized methodologies and consistent definitions for key outcomes, such as vaccination completion, are critical for improving comparability across studies (CDC 2023). The use of electronic health records and other objective data sources can enhance the accuracy and reliability of findings. Longitudinal studies are needed to explore trends over time and assess the impact of interventions on vaccination rates. Additionally, future work should investigate the role of regional healthcare infrastructure in shaping disparities, particularly between rural and urban populations (Adjei Boakye et al. 2023; Lai et al. 2016). Evaluating the effectiveness of targeted intervention strategies, such as mobile vaccination units and school‐based programs, can provide actionable insights for reducing HPV vaccination disparities. By addressing these areas, future research can contribute to more equitable vaccination outcomes and a reduction in HPV‐related cancer burdens (Munn et al. 2019; Victory et al. 2019).

## 5 Conclusion

This review highlights that HPV vaccination uptake among adolescents is shaped by complex interactions between individual factors, healthcare provider recommendations, parental beliefs, geographic location, and policy environments. Addressing disparities in HPV vaccination rates requires comprehensive, multi‐level interventions focused on improving healthcare provider communication, increasing parental awareness, and implementing policies that support both vaccine initiation and completion. Strengthening public health initiatives targeting rural and underserved populations, normalizing HPV vaccination as part of routine adolescent care, and leveraging school‐based health centers and community‐based resources can support increased uptake. By addressing the outlined barriers, public health efforts can move closer to achieving widespread HPV vaccine coverage and ultimately reduce the incidence of HPV‐related cancers among adolescents.

## Conflicts of Interest

The authors declare no conflicts of interest.

## Supporting information


**Table S1:** Factors associated with initiation of HPV vaccination.
**Table S2:** Factors associated with completion of HPV vaccination.

## Data Availability

The data that support the findings of this study are available from the corresponding author upon reasonable request.
