# Supplementary material for: Factors Influencing HPV Vaccination Uptake in Adolescents: Evidence to Guide Clinical Practice
Source: Worldviews Evid Based Nurs. 2026 Feb 18;23(1):e70120. doi: 10.1111/wvn.70120 (PMC12917299; doi:10.1111/wvn.70120)
Supplement: Supplementary file 1 — Table S1: Factors associated with initiation of HPV vaccination. Table S2: Factors associated with completion of HPV vaccination. [file WVN-23-0-s001.docx]

Supplemental Material

*Supplementary Table S1. Factors associated with initiation of HPV vaccination*

|  | **Social Determinants of Health >>>** |  |  |  |  |  |  |  |  |  |  |  |
| --- | --- | --- | --- | --- | --- | --- | --- | --- | --- | --- | --- | --- |
| **Author and Year** | **Age** | **Gender** | **Race** | **Ethnicity** | **Geographic Location (Urban vs Rural)** | **Health Insurance** | **Household Income** | **Parental Education Level** | **Parental Vaccination Knowledge** | **Healthcare Access, Factors, and Utilization** | **Parental Health Behaviors and Attitudes** | **Provider Recommendation** |
| **AdjeiBoakye, 2023** | X+ | X+ | X+ | X+ | X+ | X+ | X+ | X |  | X+ | X+ |  |
| **AdjeiBoakye, 2017** | X+ | X+ | X+ | X+ | X+ | X+ | X+ | X |  | X+ | X+ |  |
| **Agawu, 2015** | X+ | X+ | X+ | X+ |  | X+ | X | X+ |  | X+ |  | X+ |
| **Bednarczyk, 2019** | X+ | X+ | X | X |  | X+ | X+ | X+ |  | X+ |  | X+ |
| **Bhatta, 2015** | X+ | X+ | X+ | X+ | X+ | X | X+ | X+ |  | X+ |  | X+ |
| **Clark, 2016a** |  |  |  |  |  |  |  |  |  |  | X+ |  |
| **Cullen, 2014** | X+ | X | X+ | X+ |  | X+ | X+ | X+ |  | X+ |  | X+ |
| **E jezie, 2024** | X+ | X+ | X+ | X+ |  | X+ | X+ | X+ |  | X+ | X | X+ |
| **Fuchs, 2016** |  |  |  |  |  |  |  | X |  |  | X+ |  |
| **Goodman, 2023** | X+ | X+ | X+ | X+ | X+ | X+ | X+ | X+ |  | X |  | X+ |
| **Henry, 2017** | X+ |  | X+ | X+ | X+ | X+ | X+ | X+ |  | X+ |  | X+ |
| **Jeyarajah, 2016** | X+ | X+ | X+ | X+ |  | X+ | X+ | X+ |  | X+ |  | X+ |
| **Johnson, 2017** | X+ | X+ | X+ | X+ | X | X+ | X+ | X+ |  |  |  | X+ |
| **Kepka, 2015** |  |  |  |  |  |  |  | X | X+ |  |  |  |
| **Krakow 2017** | X+ | X+ | X+ | X+ | X+ | X+ | X | X+ |  | X+ | X+ |  |
| **Lai 2016** | X+ | X | X+ | X+ | X+ | X+ | X+ | X+ |  | X+ | X+ | X+ |
| **Lu 2015** | X+ | X+ | X+ | X+ |  | X+ | X | X+ |  | X+ |  | X+ |
| **Lu 2018** | X+ | X | X+ | X+ | X+ | X+ | X+ | X+ |  | X+ |  |  |
| **Mansfield 2021** | X+ | X+ | X+ | X+ |  | X+ | X+ | X+ |  | X |  | X+ |
| **Munn 2019** |  |  |  |  |  |  |  |  | X |  |  |  |
| **Pourebrahi 2021** |  |  | X | X | X+ |  |  |  |  |  |  |  |
| **Pruitt 2022** | X+ | X+ | X+ | X+ | X | X+ | X+ | X+ |  | X+ |  |  |
| **Rahman 2017** | X+ | X+ | X+ | X+ | X | X+ | X+ | X+ |  | X+ | X |  |
| **Rahman 2015a** | X+ | X+ | X+ | X+ | X+ | X+ | X | X+ |  | X+ |  | X+ |
| **Rahman 2015b** | X+ | X+ | X+ | X+ | X+ | X | X+ |  | X+ | X+ | X+ |  |
| **Reiter 2014** | X+ | X+ | X+ | X+ | X+ | X+ | X+ | X |  | X+ |  |  |
| **Staples 2021** | X+ | X+ | X | X | X+ | X+ | X+ | X+ |  | X+ |  | X+ |
| **Staras 2021** | X+ | X | X+ | X+ | X+ | X+ | X+ | X+ |  |  |  | X+ |
| **Swiecki-Skora 2019** | X+ | X+ | X+ | X+ | X+ | X | X+ | X+ |  | X+ |  | X+ |
| **Teplow-Phipps 2016** | X+ | X+ | X+ | X+ | X+ | X+ | X+ | X+ |  |  | X | X+ |
| **Thompson 2020** | X+ | X+ | X+ | X+ | X+ | X+ | X+ | X+ |  | X |  |  |
| **Torres 2022** | X+ | X+ | X+ | X+ | X | X+ | X+ | X+ |  | X+ |  |  |
| **Varman 2018** | X+ | X+ | X | X |  | X+ | X+ | X+ |  | X+ |  | X+ |
| **White 2024** | X+ | X+ | X+ | X+ | X+ | X+ | X+ | X |  | X+ |  | X+ |
| **Yankey 2020** | X+ | X+ | X+ | X+ | X+ | X+ | X | X+ |  | X+ |  | X+ |
| **Yoo 2020** | X+ | X+ | X+ | X+ | X+ | X | X+ | X+ |  | X+ |  | X+ |

*Notes. += researcher indicates variable had a significant relationship to the outcome variables.*

*Supplementary Table S2. Factors associated with completion of HPV vaccination*

|  | **Social Determinants of Health >>>** |  |  |  |  |  |  |  |  |  |  |  |
| --- | --- | --- | --- | --- | --- | --- | --- | --- | --- | --- | --- | --- |
| **Author and Year** | **Age** | **Gender** | **Race** | **Ethnicity** | **Geographic Location (Urban vs Rural)** | **Health Insurance** | **Household Income** | **Parental Education Level** | **Parental Vaccination Knowledge** | **Healthcare Access, Factors, and Utilization** | **Parental Health Behaviors and Attitudes** | **Provider Recommendation** |
| **AdjeiBoakye, 2023** | X+ | X+ | X+ | X+ | X+ | X+ | X+ | X |  | X+ | X+ |  |
| **AdjeiBoakye, 2017** | X+ | X+ | X+ | X+ | X+ | X+ | X+ | X |  | X+ | X+ |  |
| **Agawu, 2015** | X+ | X+ | X+ | X+ |  | X+ | X | X+ |  | X+ |  | X+ |
| **Bednarczyk, 2019** | X+ | X+ | X | X |  | X+ | X+ | X+ |  | X+ |  | X+ |
| **Bhatta, 2015** | X+ | X+ | X+ | X+ |  | X | X+ | X+ |  | X+ |  | X+ |
| **Clark, 2016a** |  |  |  |  |  |  |  |  |  |  | X |  |
| **Clark, 2016b** |  |  |  |  |  |  |  |  |  |  | X+ |  |
| **Cullen, 2014** | X+ | X | X+ | X+ |  | X+ | X+ | X+ |  | X+ |  | X+ |
| **E jezie, 2024** | X+ | X+ | X+ | X+ |  | X+ | X+ | X+ |  | X+ | X | X+ |
| **Fuchs, 2016** |  |  |  |  |  |  |  | X |  |  | X+ |  |
| **Goodman, 2023** | X+ | X+ | X+ | X+ | X+ | X+ | X+ | X+ |  | X |  | X+ |
| **Henry, 2017** | X+ | X+ | X+ | X+ | X | X+ | X+ | X+ |  | X+ |  | X+ |
| **Jeyarajah, 2016** | X+ |  | X+ | X+ |  | X+ | X+ | X+ |  | X+ |  | X+ |
| **Johnson, 2017** | X+ | X+ | X+ | X+ | X | X+ | X+ | X+ |  | X+ |  | X+ |
| **Kepka, 2015** |  |  |  |  |  |  |  | X | X+ |  |  |  |
| **Lai 2016** | X+ | X | X+ | X+ | X+ | X+ | X+ | X+ |  | X+ |  | X+ |
| **Lu 2015** | X+ | X+ | X+ | X+ |  | X+ | X | X+ |  | X+ |  | X+ |
| **Lu 2018** | X+ | X+ | X | X | X+ |  | X+ | X+ |  | X+ |  |  |
| **Mansfield 2021** | X+ | X+ | X+ | X+ |  | X+ | X+ | X+ |  | X |  | X+ |
| **Munn 2019** |  |  |  |  |  |  |  |  | X |  |  |  |
| **Pourebrahi 2021** |  |  | X | X | X+ |  |  |  |  |  |  |  |
| **Pruitt 2022** | X+ | X+ | X+ | X+ | X | X+ | X+ | X+ |  | X+ |  |  |
| **Rahman 2017** | X+ | X+ | X+ | X+ | X | X+ | X+ | X+ |  | X+ | X |  |
| **Rahman 2015a** | X+ | X+ | X+ | X+ |  | X+ | X | X+ |  | X+ |  | X+ |
| **Rahman 2015b** | X+ | X+ | X+ | X+ |  | X | X+ | X+ |  | X+ | X+ | X+ |
| **Reiter 2014** | X+ | X+ | X+ | X+ | X+ | X+ | X+ | X |  |  | X+ | X+ |
| **Staples 2021** | X+ | X+ | X | X | X+ | X+ | X+ | X+ |  | X+ |  | X+ |
| **Staras 2021** | X+ | X | X+ | X+ | X+ | X+ | X+ | X+ |  | X+ |  | X+ |
| **Swiecki-Skora 2019** | X+ | X+ | X+ | X+ | X+ | X | X+ | X+ |  | X+ |  | X+ |
| **Teplow-Phipps 2016** | X+ | X+ | X+ | X+ | X+ | X+ | X+ | X+ |  | X+ | X | X+ |
| **Thompson 2020** | X+ | X+ | X+ | X+ | X+ | X+ | X+ | X+ |  | X |  |  |
| **Torres 2022** | X+ | X+ | X+ | X+ | X | X+ | X+ | X+ |  | X+ |  |  |
| **Varman 2018** | X+ | X+ | X | X |  | X+ | X+ | X+ |  | X+ |  | X+ |
| **White 2024** | X+ | X+ | X+ | X+ | X+ | X+ | X+ | X |  | X+ |  | X+ |
| **Yankey 2020** | X+ | X+ | X+ | X+ | X+ | X+ | X | X+ |  | X+ |  | X+ |
| **Yoo 2020** | X+ | X+ | X+ | X+ | X+ | X | X+ | X+ |  | X+ |  | X+ |

*Notes. += researcher indicates variable had a significant relationship to outcome variables.*
